# Ectopic Overexpression of Histone H3K4 Methyltransferase CsSDG36 from Tea Plant Decreases Hyperosmotic Stress Tolerance in *Arabidopsis thaliana*

**DOI:** 10.3390/ijms22105064

**Published:** 2021-05-11

**Authors:** Qinghua Chen, Linghui Guo, Yanwen Yuan, Shuangling Hu, Fei Guo, Hua Zhao, Zhenyu Yun, Yu Wang, Mingle Wang, Dejiang Ni, Lin Zhao, Pu Wang

**Affiliations:** 1 Key Laboratory of Horticultural Plant Biology, Ministry of Education, College of Horticulture and Forestry Sciences, Huazhong Agricultural University, Wuhan 430070, China; cqhmail@webmail.hazu.edu.cn (Q.C.); Glh@webmail.hazu.edu.cn (L.G.); yuanyanwen@webmail.hazu.edu.cn (Y.Y.); hushuangling@webmail.hzau.edu.cn (S.H.); guofei@mail.hzau.edu.cn (F.G.); zhaohua@mail.hzau.edu.cn (H.Z.); catea37@mail.hzau.edu.cn (Y.W.); wangmingle@mail.hzau.edu.cn (M.W.); nidj@mail.hzau.edu.cn (D.N.); 2 Key Laboratory of Urban Agriculture in Central China, Ministry of Agriculture, College of Horticulture and Forestry Sciences, Huazhong Agricultural University, Wuhan 430070, China; 3 China National Institute of Standardization, Beijing 100191, China; yunzy@cnis.ac.cn

**Keywords:** *Camellia sinensis*, *SDG36*, histone methylation, hyperosmotic stress

## Abstract

Histone methylation plays an important regulatory role in the drought response of many plants, but its regulatory mechanism in the drought response of the tea plant remains poorly understood. Here, drought stress was shown to induce lower relative water content and significantly downregulate the methylations of histone H3K4 in the tea plant. Based on our previous analysis of the SET Domain Group (*SDG*) gene family, the full-length coding sequence (CDS) of *CsSDG36* was cloned from the tea cultivar ‘*Fuding Dabaicha*’. Bioinformatics analysis showed that the open reading frame (ORF) of the *CsSDG36* gene was 3138 bp, encoding 1045 amino acids and containing the conserved structural domains of PWWP, PHD, SET and PostSET. The CsSDG36 protein showed a close relationship to AtATX4 of the TRX subfamily, with a molecular weight of 118,249.89 Da, and a theoretical isoelectric point of 8.87, belonging to a hydrophilic protein without a transmembrane domain, probably located on the nucleus. The expression of *CsSDG36* was not detected in the wild type, while it was clearly detected in the over-expression lines of Arabidopsis. Compared with the wild type, the over-expression lines exhibited lower hyperosmotic resistance by accelerating plant water loss, increasing reactive oxygen species (ROS) pressure, and increasing leaf stomatal density. RNA-seq analysis suggested that the *CsSDG36* overexpression caused the differential expression of genes related to chromatin assembly, microtubule assembly, and leaf stomatal development pathways. qRT-PCR analysis revealed the significant down-regulation of stomatal development-related genes (*BASL*, *SBT1.2(SDD1)*, *EPF2*, *TCX3*, *CHAL*, *TMM*, *SPCH*, *ERL1*, and *EPFL9*) in the overexpression lines. This study provides a novel sight on the function of histone methyltransferase CsSDG36 under drought stress.

## 1. Introduction

With the rapid development of plant epigenetics, the regulatory mechanisms of plant histone modification in various biological processes have become hot research topics. Histone modification has been shown to affect the structure and condensation state of chromatin by altering the way histones bind to DNA [1]. The genome-wide distribution of various histone modifications has been reported in *Arabidopsis* [2], rice [3] and corn [4], and SDG (SET domain group) is identified as the only protein family with histone methyltransferase activity in plants. In *Arabidopsis thaliana*, SET structural analysis showed seven types of SET domain-containing proteins: (E(Z) family, ASH1 family, Trithorax (TRX) family—proteins containing SET and PHD domains, and Su(var) family—proteins containing interrupted SET domains, non-histone methyltransferases and similar proteins [5]. The TRX subfamily members catalyze the methylation of histone H3K4 and functions near the transcription start site [6]. In *Arabidopsis thaliana*, the TRX subfamily has been reported to have five Trithorax (*ARABIDOPSIS TRITHORAX1-5 (ATX1-5)*) and seven Trithorax-related genes (*ATX-RELATED1-7 (ATXR1-7)*) [7,8,9].

In plants, histone modifications have been shown to be involved in many biological processes, such as flower development [10,11], seed germination, root organ growth and development, regulation of the biological clock and the number of side branches [12,13,14,15], plant morphogenesis [16], and stress response [17,18]. In *Arabidopsis* thaliana, ATX1 has been reported to regulate many biological processes via H3K4me3 methylation, including cell wall modifying [19], flowering [17], and dehydration stress response through ABA-dependent and non-ABA-dependent pathways [18]. Drought response genes *RD29A* and *RD20* in *Arabidopsis thaliana* are related to the increase in H3K4me3 and H3K9ac activities under drought stress, leading to the disappearance of nucleosomes and affecting the chromatin state [20,21]. In *Arabidopsis thaliana,* the H3K4me1, H3K4me2 and H3K4me3 levels on all the genes were significantly varied under drought stress [22]. Previous studies have shown that AtSDG8 can catalyze the di-methylation and tri-methylation of H3K36 and the trimethylation of H3K9, regulating shoot restructuring [12], FLOWERING LOCUS C (*FLC*)-mediated flowering [23], and sensitivity to light and carbon signals [24]. In rice, the H3K4me3 levels on 4837 genes were modified under drought stress [3]. As reported previously, OsSDG725 can catalyze the di-methylation and trimethylation of histone H3K36 and regulate the synthesis of steroids or signaling pathways of brassinosteroids, introns marks, and RNA splicing [25,26]; OsSDG724 can catalyze the methylation and trimethylation of histone H3K36 and regulate flowering in rice [27]; OsSDG714 can catalyze the di-methylation of histone H3K9 and regulate rice DNA methylation, affecting genomic stability [28,29]. In barley (*Hordeum Vulgare* L.), the histone H3K4 methyltansferase gene was reported to regulate seed growth and development, with a specific inducing effect on drought stress [30].

In recent years, the tea plant (*Camellia sinensis* (L.) O. Kuntze), an important perennial leaf economic crop, has been shown to be susceptible to drought stress, characterized by leaf aging and shedding, decrease in the content of secondary metabolites in new shoots, and blockage in the synthesis pathways of catechins, caffeine and amino acids in tea leaves [31]. Additionally, Z-3-hexenal has been reported to activate the *DREB* and *RD* gene expression in tea plants and positively regulate the *LOXs* and *ADHs* genes to increase tea plant tolerance to hyperosmotic stress [32]. In the tea plant, the *CsCOR1* gene regulates its dehydration tolerance by modifying cell walls and the ABA-dependent and ABA-independent pathways [33], and the *CsLEA7* gene positively regulates tolerance to drought stress [34]. Under hyperosmotic stress, miR159 is significantly upregulated, which activates *ABI3* expression through an ABA-dependent pathway, thus enhancing its osmotic tolerance [35,36]. In our previous study, the *SDG* genes were identified and CsSDG36 was clustered into the TRX subfamily, which catalyze H3K4me2 and H3K4me3 in vivo [37]. However, the function of CsSDG36 remains unclear.

The purpose of this study was to explore the function of the *CsSDG36* gene under drought stress. To this end, the relative water content and histone methylation levels were analyzed under hyperosmotic stress. Based on our previous study, the full-length coding sequence (CDS) of *CsSDG36* was cloned from the tea cultivar ‘Fuding Dabaicha’, followed by bioinformatics analysis and hyperosmotic function analysis using *Arabidopsis thaliana* as a model to explore the function of CsSDG36. Compared with wild-type Arabidopsis, *CsSDG36* over-expression lines exhibited lower hyperosmotic resistance by accelerating plant water loss, increasing reactive oxygen species (ROS) pressure, and increasing leaf stomatal density. RNA-seq analysis suggested that CsSDG36 protein is associated with the chromatin assembly, microtubule assembly, and leaf stomatal development pathways. qRT-PCR analysis revealed a significant downregulation in the stomatal development-related genes (*BASL, SBT1.2(SDD1)*, *EPF2*, *TCX3*, *CHAL*, *TMM*, *SPCH*, *ERL1*, and *EPFL9*) in overexpression lines.

## 2. Results

### 2.1. Histone H3K4 Methylation in Tea Leaves Is Significantly Down-Regulated under Drought Stress

**Figure 1:**
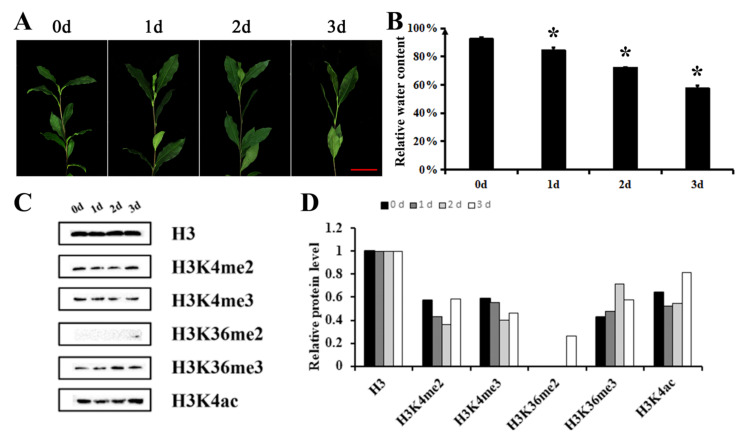
Histone methylation analysis of tea leaves in response to hyperosmotic treatment (20% PEG 6000) for Days 0, 1, 2 and 3. (**A**) Images of tea leaves after hyperosmotic treatment. Bars = 50 mm. (**B**) Relative water content of tea leaves after hyperosmotic treatment. (**C**) Histone modifications of lysine 4 and lysine 36 on histone H3 protein. H3K4me2: Di-methylation of lysine 4 on histone H3 protein. H3K4me3: Tri-methylation of lysine 4 on histone H3 protein. H3K36me2: Di-methylation of lysine 36 on histone H3 protein. H3K36me3: Tri-methylation of lysine 36 on histone H3 protein. H3K4ac: Acetylation of lysine 4 on histone H3 protein. H3 protein is the loading control. (**D**) Semi-quantitative analyses of western blot results in (**C**). The protein content was determined by normalizing the band intensity of specific antibodies using the software IMAGEJ. * represents significant difference at *p* < 0.05 versus the 0d sample. The results were obtained from three biological experiments with three technical repetitions.

In order to detect the histone methylation levels of tea plants under hyperosmotic stress, tea plants were treated with 20% PEG6000 for three days. Tea leaves became wilted and the relative water content decreased after hyperosmotic treatment (Figure 1A,B). Compared with Day 0, the H3K4me2 and H3K4me3 levels in the tea seedling leaves showed a decrease on Day 1 of dehydration, followed by a decrease to the lowest level on Day 2, and then a slight recovery on Day 3 of dehydration (Figure 1A,B). Meanwhile, the H3K36me2 level increased with dehydration time, and the H3K36me3 level showed a steady increase and reached the highest level on Day 2 of dehydration, followed by a decrease, but was higher than that of the untreated tea seedlings (Day 0) on Day 3. The H3K4ac level decreased first and then increased and was higher on Day 3 than that of tea seedlings without dehydration (Day 0). The decrease in H3K4me2 and H3K4me3 levels suggested the potential roles of TRX members in hyperosmotic stress tolerance in *Camellia sinensis*, including CsSDG36 protein.

### 2.2. Amino Acid Sequence Analysis of CsSDG36 Gene

**Figure 2:**
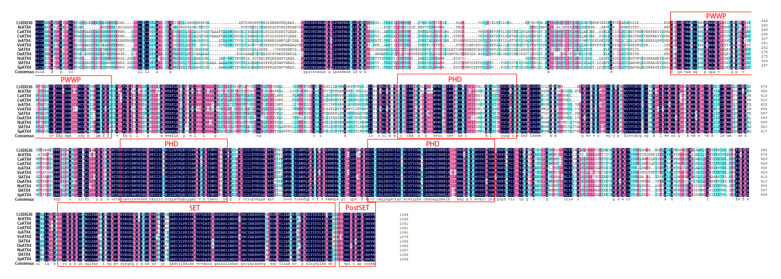
Multiple sequence alignment of CsSDG36 and ATX4 proteins from other species. Conserved domains of ATX4 proteins were marked with red boxes, including one PWWP, three PHD, one SET, and one PostSET domains.

According to the data of the *SDG* gene family in our previous study, the full-length SDS sequence of *CsSDG36* was cloned from tea cultivar ‘Fuding Dabaicha’ [37]. The amino acid sequence of the *CsSDG36* gene was analyzed by multiple comparisons with highly similar proteins in the NCBI database, which is highly similar to ATX4 proteins of different species containing the amino acid of PWWP, PHD, SET and PostSET domains (Figure 2). The amino acid sequence of CsSDG36 was analyzed by ExPASy ProtParam and the results are shown in Table 1. The CsSDG36 protein was shown to contain 1045 aa, with a relative molecular mass of 118,249.89 Da, a theoretical isoelectric point (PI) of 8.87, 121 positively changed residues (Asp + Glu), 153 negatively charged residues (Arg and Lys), an unstable coefficient of 45.41 (unstable), an aliphatic amino acid content of 85.5%, an aromatic amino acid content of 8.0%, a heterocyclic amino acid content of 6.5%, an acidic amino acid content of 11.6%, and a basic amino acid content of 16.9%. The total average hydrophilicity (GRAVY) is −0.440, indicating that CsSDG36 is a hydrophilic protein. Meanwhile, the hydrophilic and hydrophobic map of the protein was drawn by the online tool ProtScale (Figure S1), and the results showed a higher ratio of hydrophilic amino acid residues than hydrophobic amino acid residues, suggesting that CsSDG36 is a hydrophilic protein, which is consistent with the amino acid sequence analysis of the protein. TM-HMM Server 2.0 was used to predict the transmembrane structure of the protein and the results showed that the protein did not cross the membrane. Additionally, the online tool Cell-PLOc 2.0 was used to predict the subcellular localization and CsSDG36 was shown to be localized in the nucleus.

### 2.3. Phenotypic Profiling of CsSDG36 Protein in Arabidopsis thaliana

**Figure 3:**
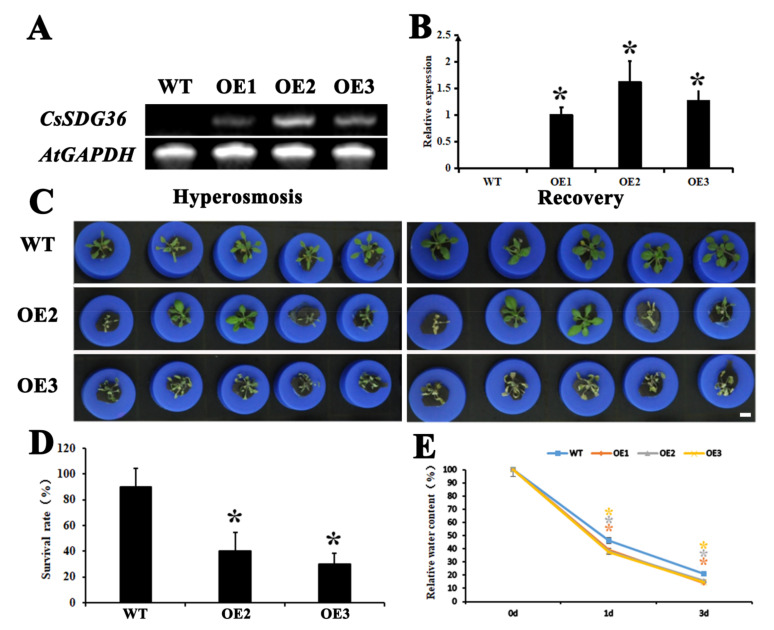
Phenotypic identification of *CsSDG36* in hyperosmosis between Arabidopsis seedlings of overexpression lines and wild type. (**A**) Semi-quantitative PCR analysis of *CsSDG36* expression in overexpression lines. (**B**) qRT-PCR analysis of *CsSDG36* expression in overexpression lines. (**C**) Seedlings after 6-day hyperosmotic treatment (10% PEG 6000) and 2-day recovery (1/4 nutrient solution). (**D**) Survival rates of seedlings after recovery. (**E**) Relative water contents of seedling leaves after hyperosmotic treatment. WT, wild type; OE1, overexpression line 1; OE2, overexpression line 2; OE3, overexpression line 3. Bars = 10 mm. * represents significant difference at *p* < 0.05 versus the wild type. The results were obtained from three biological experiments with three technical repetitions.

The full-length coding sequence (CDS) of *CsSDG36* was transformed into wild-type (WT) Arabidopsis, and three homozygous overexpression lines were screened out for phenotypic identification. Compared with the wild type (Figure 3A,B), CsSDG36 was significantly expressed in the three overexpression lines, and lines 2 and 3 were selected for subsequent identification. Arabidopsis seedlings were cultured for 20 days, followed by 6 days of treatment with 10% PEG 6000, and 2 days of restoration in 1/4 of the nutrient solution. The overexpression lines were found to be more sensitive to hyperosmotic stress than wild-type *Arabidopsis thaliana* (Figure 3C). The survival rate was significantly lower in the overexpression lines than in the wild type (Figure 3D), and the relative water content was also significantly lower in the overexpression lines than in the wild type after hyperosmotic treatment (Figure 3E). The dry plant weight was significantly higher for over-expression lines than the wild type after 4-week normal cultivation (Figure S2).

**Figure 4:**
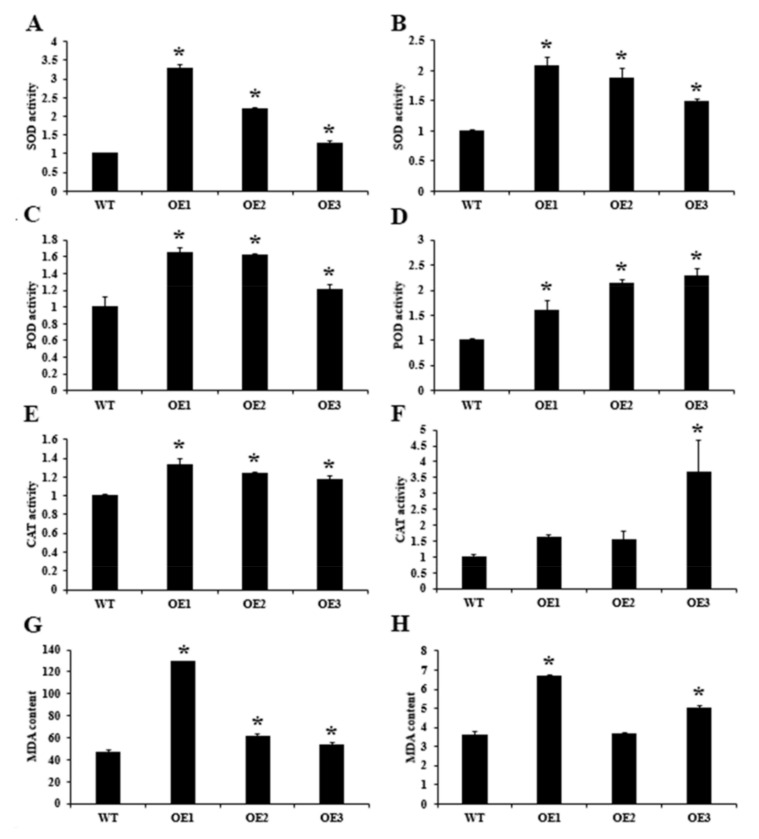
Relative ROS-related enzyme activities and MDA contents in Arabidopsis seedlings after 3 days of hyperosmotic treatment (10 % PEG 6000). (**A**) Relative SOD activity in leaves. (**B**) Relative SOD activity in roots. (**C**) Relative POD activity in leaves. (**D**) Relative POD activity in roots. (**E**) CAT content in leaves. (**F**) CAT content in roots. (**G**) MDA content in leaves. (**H**) MDA content in roots. WT, wild type; OE1, overexpression line 1; OE2, overexpression line 2; OE3, overexpression line 3. * represents significant difference at *p* < 0.05 versus the wild type. The results were obtained from three biological experiments with three technical repetitions.

**Figure 5:**
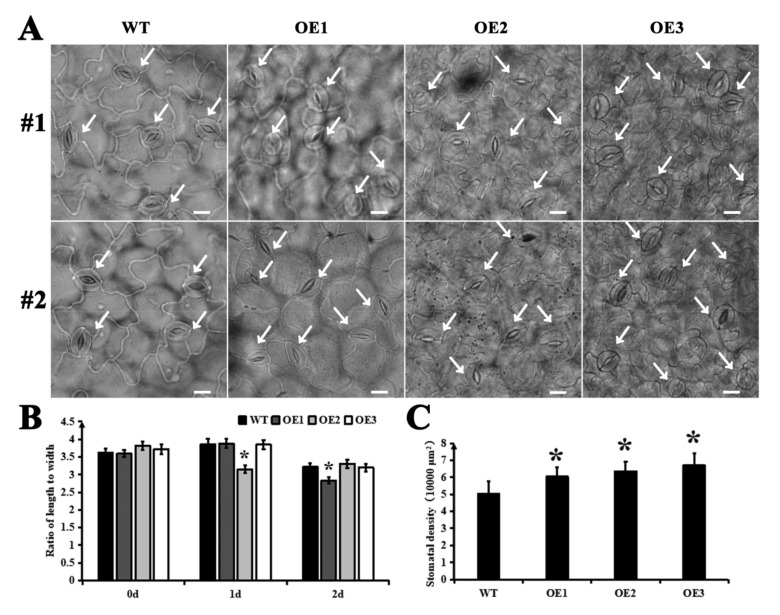
Stomatal density, opening degree of lower epidermis leaves in Arabidopsis seedlings after hyperosmotic treatment (10% PEG 6000). (**A**) The in situ detection of stomatal distribution on the lower epidermis of WT, OE1, OE2 and OE3 leaves via light microscope. #1 and #2 represent different fields of vision. The white arrows indicate stomatal. Per unit area 1 × 10^4^ μm^2^. Bars = 10 μm. (**B**) Stomatal closure quantified by measuring the length to width ratios of stomata in a triplicate experiment with 100 pairs of guard cells per experiment. (**C**) Stomatal distribution density in the lower epidermis of Arabidopsis leaves. WT, wild type; OE1, overexpression line 1; OE2, overexpression line 2; OE3, overexpression line 3. * represents significant difference at *p* < 0.05 versus the wild type. The results were obtained from three biological experiments with three technical repetitions.

By measuring the activity of SOD, POD, CAT and other related enzymes, the physiological states of overexpression lines and wild type under high osmotic stress were compared. After 3 days of high osmotic stress treatment, the related enzyme activities were significantly higher in overexpression plants than in the wild-type plants (Figure 4A–F). The MDA content was further measured to compare the redox status of the overexpression and wild-type strains under hyperosmotic stress. After 3 days of hyperosmotic treatment, the MDA content was significantly higher in overexpressed leaves and roots than in wild-type leaves and roots (Figure 4G,H).The stomatal conditions of Arabidopsis thaliana leaf epidermis were observed under the bright field of a positive fluorescence microscope (DM6BDM6B, Leica) (Figure 5A). The stomatal cells exhibited little difference in appearance, but some difference in stomatal number between over-expression lines and wild type. The stomatal length-to-width ratios in different fields of view were counted to compare the changes in the stomatal openings of overexpression and wild-type strains under hyperosmotic treatment (10% PEG 6000), with no significant difference being observed in stomatal opening size between over-expression and wild-type strains (Figure 5B). Stomatal density was analyzed by counting the stomatal number in different fields. Meanwhile, stomatal number was compared between over-expression and wild-type lines under the same area (1 × 10^4^ μm^2^). As shown in Figure 5C, the stomatal density was significantly (*p* < 0.05) higher in the three overexpression lines than in the wild-type line. The increase in stomatal density accelerates leaf water loss.

### 2.4. CsSDG36 Protein Is Associated with the Chromatin Assembly, Microtubule Assembly, and Stomatal Development Pathways

**Figure 6:**
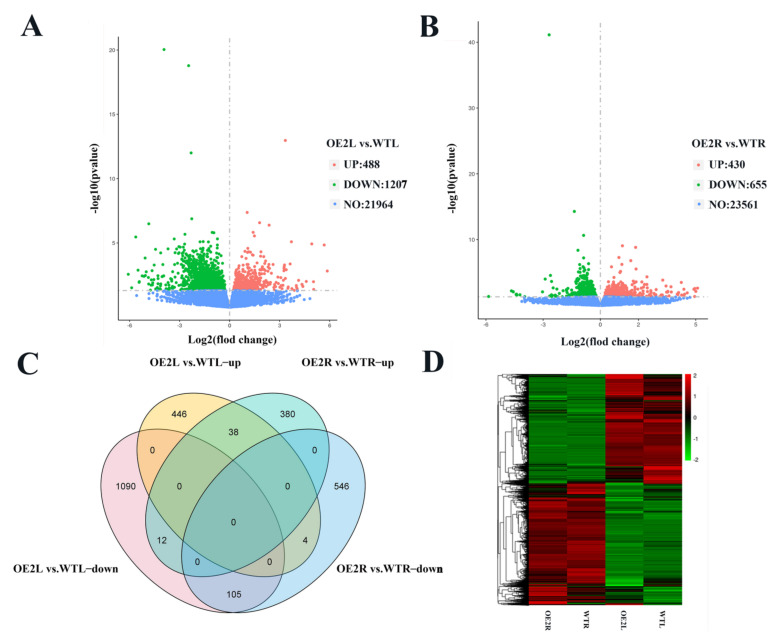
The landscape of *CsSDG36* transcriptional regulation in *Arabidopsis thaliana*. (**A**) Filter-Volcano plot of overexpression lines vs. the wild-type line in leaves (log2 (fold change)). (**B**) Filter–Volcano plot of overexpression lines vs. the wild-type line in roots (log2 (fold change)). (**C**) Venn analysis of overexpression lines vs. the wild-type line in leaves and roots about downregulated genes (DRGs) and upregulated genes (URGs). (**D**) Hierarchical clustering analyses of differentially expressed genes (DEGs): downregulated genes (DRGs) and upregulated genes (URGs) between wild-type (WT) and overexpression line 2 in leaves and roots. Heat color gradation in red and green denotes an increase and a decrease, respectively. OE2, overexpression line 2. Error bars indicate mean ± SD (*n* = 3). The results were obtained from three biological experiments.

Statistics, quality control, assembly analysis, and functional annotation were performed for the raw transcriptome data. The differentially expressed genes (DEGs) in the transcripts were screened by a threshold padj < 0.05. A total of 1695 DEGs were screened between overexpression line 2 and wild-type leaves, including 488 upregulated and 1207 downregulated genes (Figure 6A). Meanwhile, 1085 DEGs were obtained between overexpression line 2 and wild-type roots, including 430 upregulated and 655 downregulated genes (Figure 6B). In the Venn diagram (Figure 6C), the over-expression and wild-type lines showed the overlap between leaf and root differential genes, with 38 overlap DEGs upregulated and 105 overlap DEGs downregulated. In the leaf, 446 and 1090 DEGs were respectively up- and downregulated, while in the root, 380 and 546 DEGs were respectively up- and downregulated. Cluster analysis of all the DEGs showed tissue specificity in the leaf and root gene expression between overexpression and wild-type lines (Figure 6D).

**Figure 7:**
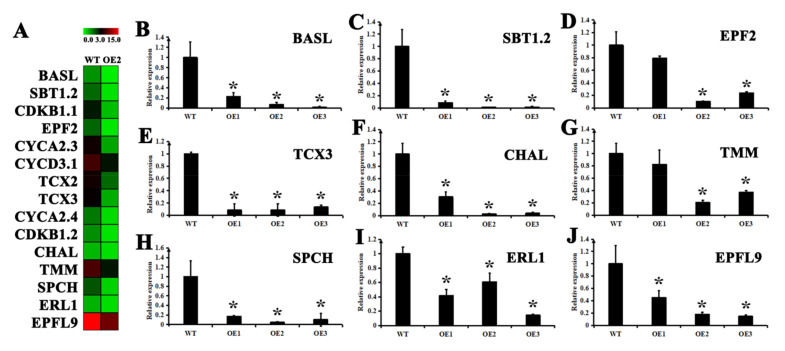
The landscape of *CsSDG36* transcriptional regulation in *Arabidopsis thaliana*. (**A**) Hierarchical clustering analysis of genes in the categories ‘response to Stomatal development’. (**B**–**J**) Quantitative RT-PCR analysis of genes in the categories ‘response to Stomatal development’. Glyceraldehyde-3-phosphate dehydrogenase (*GAPDH*) was used as an internal control. Error bars indicate mean ± SD (*n* = 3). * represents significant difference at *p* < 0.05 versus the wild type. The results were obtained from three biological experiments with three technical repetitions.

The GO enrichment analysis showed that DEGs were mainly enriched in cytoskeletal and microtubules (Figure S3A, Table S1). In the whole metabolic process of leaves, over 1600 DEGs were upregulated or downregulated, and 600 of them were related to cytoskeleton and microtubule cells, indicating that the stomata of the leaves are closed under dehydration conditions. Additionally, GO analysis revealed the enrichment of DEGs mainly in “Chromatin Assembly”, “RNA synthesis” and “oxidoreductase activity” (Figure S3B, Table S2). During the entire root metabolism process, over 1000 DEGs were upregulated or downregulated, and over 150 of them were related to chromatin assembly, RNA synthesis, and redox enzyme activity. The CsSDG36 protein regulates histone methylation and indirectly affects chromatin assembly and RNA synthesis, while the increase in redox enzyme activity is also consistent with the significantly higher enzyme activity of the overexpression strain than the wild-type strain after hypertonic treatment. The 15 genes related to vent hole development in wild type and overexpression line 2 include: *BASL (BREAKING OF ASYMMETRY IN THE STOMATAL LINEAGE), SBT1.2 (SDD1;STOMATAL DENSITY AND DISTRIBUTION 1), CDKB1-1 (CYCLIN-DEPENDENT KINASE B1;1), EPF2 (EPIDERMAL PATTERNING FACTOR 2), CYCA2-3 (CYCLIN A2;3), CYCD3-1 (CYCLIN D3;1), TCX2 (TESMIN/TSO1-LIKE CXC 2), TCX3 (CXC domain containing TSO1-like protein 1), CYCA2-4 (CYCLIN A2;4), CDKB1-2 (CYCLIN-DEPENDENT KINASE B1;2), CHAL (EPFL6,EPIDERMIS PATTERNING FACTOR (EPF1)-LIKE 6), TMM (TOO MANY MOUTHS;ATRLP17;RECEPTOR LIKE PROTEIN 17), SPCH (SPEECHLESS), ERL1 (ERECTA-LIKE 1), EPFL9 (EPIDERMAL PATTERNING FACTOR LIKE-9;STOMAGEN)* (Figure 7A). These 15 genes were all downregulated and 9 genes were randomly selected for qRT-PCR verification. As shown by the qRT-PCR results in (Figure 7B–J), the stomatal development related genes (*BASL*, *SBT1.2*, *EPF2*, *TCX3*, *CHAL*, *TMM*, *SPCH*, *ERL1* and *EPFL9*) were significantly downregulated in overexpression line 2 and 3, while *EPF2* and *TMM* were not significantly changed in overexpression line 1.

## 3. Discussion

In our study, drought stress was found to significantly downregulate the methylation of histone H3K4 and upregulate its acetylation (Figure 1). Based on our previous analysis of the *SDG* gene family, the full-length CDS of *CsSDG36* was cloned from the tea variety ‘Fuding Dabaicha’. Amino acid sequence analysis revealed the protein encoded by the *CsSDG36* gene as a hydrophilic and non-transmembrane transport protein, with the conserved structural domains of PWWP, PHD, SET and PostSET. The *CsSDG36* gene belongs to the TRX subfamily, which has the activity of catalyzing the methylation of histone H3K4 [38]. Homology analysis showed that the CsSDG36 protein and Arabidopsis AtATX4 had similar conserved domains (Figure 2). It has been confirmed that the TRX subfamily in Arabidopsis, especially the ATX proteins, has the conserved structural domains of PWWP, PHD, SET and PostSET [7]. In maize, histone H3K4 methylation is conserved in chromosome distribution among five cultivation and two wild-type varieties [4]. Phylogenetic analysis of 22 SET domain proteins from maize and 32 SET domain proteins from Arabidopsis revealed that proteins containing the PWWP, PHD, SET, PostSET domains all belong to the third category of theSDG family [38], which is consistent with the results of CsSDG36 in the present study. In rice, the NCBI conserved domain database was used to determine the conserved domains and compare them with the SDG family genes of *Arabidopsis thaliana* and maize. SDG721 and SDG715 were shown to belong to the third category of the SDG family, including the conserved domains of PWWP, PHD, SET and PostSET [5], which was consistent with the ATX family of *Arabidopsis thaliana* and the results of the present study.

In plants, SDG protein can dynamically regulate chromatin condensation by mediating histone methylation on lysine residues [39]. SDG-mediated histone methylation is involved in many biological processes, such as flower organ development, root organ growth and development, and plant response to abiotic stress. The functions of TRX genes in Arabidopsis have been extensively studied, such as plant growth and development [40,41], cell wall modification [19], and drought stress response [17,18]. ATX1 catalyzes histone H3K4me3 and responds to dehydration stress through ABA-dependent and ABA-independent pathways [17]. The atx1 Arabidopsis mutation showed reduced germination rate and enlarged stomatal aperture to reduce drought tolerance [17]. In this study, the histones H3K4me2 and H3K4me3 in tea plants were significantly downregulated under drought stress, and the conserved domain of the CsSDG36 protein also indicated its close genetic relation to Arabidopsis ATX4, indicating the similarity between CsSDG36 in the present study and ATX4 and ATX5 in *Arabidopsis thaliana* in the mode of regulating dehydration stress response. Physiologically, the *CsSDG36* overexpression strains attenuated the hyperosmotic tolerance of *Arabidopsis thaliana* by increasing stomatal density, accelerating water loss, and increasing ROS pressure (Figure 3, Figure 4 and Figure 5).

RNA-seq analysis suggested that *CsSDG36* overexpression strain 2 significantly altered the growth and metabolism in the leaves and roots of *Arabidopsis thaliana*, which may play a crucial role in drought stress response (Figure S3). The GO analysis showed that the DEGs related to the leaf are mainly concentrated in a series of synthetic pathways, such as the microtubule and cytoskeleton (Figure S3A, Table S1). The DEGs related to the root are also mainly enriched in a series of synthetic pathways, such as chromatin assembly, RNA synthesis, and oxidoreductase activity. Attention should be paid to the downregulated DEGs, which may play a key role in drought stress response (Figure S3, Table S2). Roots and leaves vary in their functions in response to drought stress, and the cytoskeleton and microtubules in leaves are closely related to stomatal development and stomatal density distribution. Some studies have defined the plant water use efficiency (WUE) as the ratio between plant photosynthetic rate and transpiration rate. *GTL1* in *Arabidopsis thaliana* is a negative regulator of *SDD1* (stomatal density and distribution), which regulates its stomatal density, water and carbon dioxide absorption, and finally affects its water use efficiency [42]. In this study, CsSDG36 is a negative regulator of genes related to stomatal development, and the stomatal number was significantly higher in the leaf epidermis of the CsSDG36 overexpression line versus wild-type line, which accelerated the *Arabidopsis thaliana* transpiration rate and water loss, leading to a decrease in its water use efficiency, and a lower survival rate of CsSDG36 overexpression lines than the wild-type line under drought conditions (Figure 6). The *TaGT2L 1D* overexpression strain in wheat was shown to reduce the drought tolerance by increasing the stomatal number [43], which is consistent with the observation in this study. SDG regulates chromatin formation by mediating the activity of histone H3, and the root of *CsSDG36* overexpression line 2 showed differential gene GO enrichment in the chromatin assembly and RNA synthesis pathways, which is also attributed to the function of SDG protein. This further indicated that the leaf position of *CsSDG36* overexpression line 2 could reduce drought tolerance by regulating the stomatal number, while the root could change the chromatin composition to reduce drought tolerance. In *Arabidopsis thaliana*, ATX4 and ATX5 catalyze the histones H3K4me2 and H3K4me3 to negatively regulate the dehydration stress response through an ABA-dependent pathway regulating stomatal closure [18]. However, there was no significant differences in stomatal length-to-width ratios between wild-type and overexpressed strains under drought stress, but there was a significant difference in stomatal density. Some studies have also shown that *AtATX4* and *AtATX5* regulate the dehydration stress response by regulating key downstream genes (*AHG3*, *CYP707A2*, *HB-7*, and *RD20*) [18]. However, RNA sequencing analysis showed no significant differences between these four key downstream genes in the present study. qRT-PCR analysis revealed a significant downregulation of the stomatal development-related genes (*BASL*, *SBT1.2(SDD1)*, *EPF2*, *TCX3*, *CHAL*, *TMM*, *SPCH*, *ERL1*, and *EPFL9*) in overe-xpression lines. In Arabidopsis thaliana, *TMM* (*Too Many Mouth*) [44] and *SDD1* [45,46] are two negative regulators of stomatal development. *EPF2* plays a key role in the regulation of stomatal density, the overexpression of which promotes stomatal development [47]. In maize, overexpression of *SDD1* leads to decreased stomatal density and improved drought tolerance [48,49].

This study not only demonstrates that drought stress significantly downregulates the methylation of histone H3K4 in the tea plant, but also provides evidence that *CsSDG36* overexpression significantly reduces the hyperosmotic stress tolerance of *Arabidopsis thaliana*. The *CsSDG36* overexpression is associated with increased leaf stomatal density, reduced water use efficiency, and increased ROS pressure. The RNA-seq and qRT-PCR results showed that CsSDG36 protein is associated with the chromatin assembly, microtubule assembly, and stomatal development pathways. This study has provided a novel sight for analyzing the function of CsSDG36 protein under drought stress.

## 4. Materials and Methods

### 4.1. Plant Materials and Treatment

One-year tea seedling [*Camellia sinensis* (L.) O. Kuntze cv. *Echa 10*] was used for hyperosmotic stress treatment. Seedlings were pre-grown for 2 months in a basic solution ((NH_4_)_2_SO_4_ (0.25 mM), NH_4_NO_3_ (0.5 mM), KH_2_PO_4_ (0.5 mM), K_2_SO_4_ (0.2 mM), Ca(NO_3_)_2_ (0.5 mM), MgSO_4_ (0.8 mM), Al_2_(_S_O_4_)_3_ (0.08 mM), NaFeEDTA (35.0 μM), H_3_BO_3_ (23.0 μM), MnSO_4_ (4.5 μM), CuSO_4_ (0.15 μM), ZnSO_4_ (1.0 μM) and Na_2_MoO_4_ (0.05 μM)) [32], with the solution being refreshed once a week for 2 months. Plants were grown in a greenhouse, with a 300–320 μmol m^−2^ s^−1^ photon flux density for 12 h per day and day/night temperature of 28/24 °C. Hyperosmotic treatment was performed by 20% PEG6000 dissolved in the basic solution and samples were collected after 0-, 1-, 2- and 3-day exposure. The *CsSDG36* gene was cloned from *Fuding Dabaicha* tea seedling (30°28′0″ N, 114°22′8″ E) in the germplasm resources nursery of Huazhong Agricultural University (Wuhan, China).

### 4.2. Western Blotting

The same amount of total protein of tea leaves was extracted with the protein extraction solution (66.7 mmol/L tris-base, 2% *w*/*v* SDS, 5% *w*/*v* PVP-10, 1 mmol/L PMSF, 100 mmol/L DTT, PH = 6.8) for Western blotting analysis. Protein samples were detected by SDS-PAGE, transferred to a 0.22 μm PVDF membrane in a chamber cooled with ice water and then blocked with milk. Histone methylation antibodies were used to detect methylation levels, with histone H3 protein as the control group. IMAGE J software was used to normalize the intensity of a specific antibody band and determine protein amount. The results were obtained from three biological experiments with three technical repetitions.

### 4.3. Bioinformatics Analysis

The conserved domains of the amino acid sequences encoded by *CsSDG36* were analyzed by NCBI SMART, and the amino acid sequences of multiple species were compared by DNAMAN 6.0 (LynnonBiosoft, San Ramon, CA, USA). The composition and physicochemical properties of amino acid sequences were analyzed by the online analysis tool ProtParam (https://web.expasy.org/protparam/, accessed on 1 June 2020). The transmembrane structure of the CsSDG36 protein was predicted and analyzed by TM-HMM Server 2.0 (http://www.cbs.dtu.dk/services/TMHMM/, accessed on 1 June 2020). The hydrophilic and hydrophobic map of the protein was drawn by the online tool ProtScale (https://web.expasy.org/protscale/, accessed on 1 June 2020), and the subcellular localizations were predicted by the online tool Cell-PLOc 2.0 (http://www.csbio.sjtu.edu.cn/bioinf/Cell-PLoc-2/, accessed on 1 June 2020).

### 4.4. Overexpression of CsSDG36 in Wild-Type Arabidopsis thaliana

Based on the *CsSDG36* gene sequence obtained from the CsSDG family in our previous study [37], the complete CDS of *CsSDG36* was amplified with primers, listed in Table 1, followed by cloning the sequence into the *pCAMBIA1300s* vector and transforming the recombinant vector by agrobacterium-mediated inflorescence infection [50]. Positive screening was performed by hygromycin and PCR. CsSDG36-F and CsSDG36-R listed in Table 2 were used for the CDS cloning and positive screening, while TYXL + CsSDG36-F and TYXL + CsSDG36-R were used for the homologous recombination. The inflorescence-infected seeds were defined as T0 generation; phenotypic identification was performed using homozygous T2 generation.

### 4.5. Phenotypic Identification of CsSDG36 Over-Expression Lines

Phenotypic identification was performed using the nutrient solution culture method as reported by Conn et al. (2013). Briefly, homozygous Arabidopsis seeds were disinfected and seeded on agar, which was placed on an opaque 0.5 L box filled with nutrient solution. After 14 days of growth, the seedlings were transferred to an 8 L sealed container filled with nutrient solution, followed by 6 days of culture, 6 days of treatment with 10 % PEG 6000, and 2 days of restoration in distilled water. Finally, the survival rates of overexpression lines and wild types were calculated. For relative water content measurement, rosette leaves were completely sampled for the Arabidopsis seedling and soaked in water for 24 h, followed by drying at 80 °C for 24 h. Relative water content = [(M0 − M2)/(M1 − M2)] × 100%, where M0: fresh weight of rosette leaves; M1: weight of rosette leaves after soaking in water for 24 h; M2: weight of rosette leaves after drying at 80 °C for 24 h. The results were obtained from three biological experiments with three technical repetitions.

### 4.6. Physiological Analysis

For physiological analysis, Arabidopsis leaves and roots were ground in liquid nitrogen and dissolved in 1×PBS buffer (pH = 7.8). After centrifugation, the supernatant was collected to detect the activities of superoxide dismutase (SOD), peroxidase (POD) and catalase (CAT).

The SOD activity was determined by NBT photoreduction as reported by Beauchamp and Fridovich (1971) with slight modifications. Briefly, the enzyme solution (0.05 mL) was added to 2.95 mL reaction solution (50 mmol/L PBS, 1.25 mmol/L nitrogen tetrazolium (NBT), 0.1 mmol/L EDTA, 0.22 mol/L methionine and 33 mol/L riboflavin) for photochemical reduction reaction under 4000 lx rays for 20 min, and the OD value was obtained at 560 nm. SOD activity = [(A560CK − A560e) × V]/(A560CK × 0.5 × DW × Vt), where A560CK is the absorption value of the control substance at 560 nm; A560E, the absorption value at 560 nm; V, the total volume of crude extract; DW, the sample dry weight; and Vt, the volume of crude extract used for measurement. The results were obtained from three biological experiments with three technical repetitions.

The POD activity was determined by guaiacol assay as reported by Gajewska and Skl Odowska (2007). The enzyme solution (0.05 mL) was mixed with the reaction solution (50 mL 0.05 mol /L PBS (pH = 6.0) and 28 μL guaiacol), followed by heating to dissolve and adding 19 μL 30% H_2_O_2_ after cooling. The light absorption value was determined at 470 nm. POD activity = (ΔA470 × Vt)/(0.01 × t × Vs × DW), where ΔA470 is differences in the absorption value at 470 nm; V, total volume of crude extract; DW, sample dry weight; Vt, volume of crude extract used for measurement; and T, reaction time. The results were obtained from three biological experiments with three technical repetitions.

The CAT activity was determined by the H_2_O_2_ method as described by Okuda (1991). Briefly, the enzyme solution (0.1 mL) was added to 1.9 mL PBS (pH = 7.0) and 1 mL 0.1 mol/L H_2_O_2_ to determine the absorbance value at 240 nm. The CAT activity = (ΔA240 × Vt)/(DW 0.01 × Vs), where ΔA240 is differences in the absorption value at 240 nm; V, total volume of crude extract; DW, sample dry weight; Vt, volume of crude extract used for measurement; and T, reaction time. The results were obtained from three biological experiments with three technical repetitions.

The malondialdehyde (MDA) content was determined by spectrophotometry described by Chen et al. (2021). Briefly, the MDA in the sample was extracted with trichloroacetic acid, reacted with thiobarbituric acid (TBA) to form a pink compound, and its absorbance value at 532 nm was determined and compared with the standard. The MDA content = c × V/m where c is the MDA concentration calculated from the absorbance value at 532 nm and standard curve; V, volume of sample solution; and DW, sample dry weight. The results were obtained from three biological experiments with three technical repetitions.

### 4.7. Statistical Analysis of Stomatal Density

The stomatal number was analyzed using the nutrient solution culture method as reported by Conn et al. (2013). Briefly, homozygous Arabidopsis seeds were disinfected and seeded on agar, which was placed on an opaque 0.5 L box filled with nutrient solution. After 14 days of growth, the seedlings were transferred to an 8 L sealed container filled with nutrient solution and cultured for 6 days, followed by 2 days of treatment with 10% PEG 6000. The leaves of *Arabidopsis thaliana* were sampled every day. Finally, the untreated and treated *Arabidopsis thaliana* leaves were fixed with 1% glutaraldehyde fixative solution, and chlorinated trichloroacetaldehyde was used to facilitate the observation of leaf stomata. Under a full dynamic normal fluorescence microscope, the stomatal number in the leaf lower epidermis was observed and the number per unit area was counted. A minimum of 10 fields of view were counted for each plant line, and the stomatal length-width ratio was counted for at least 50 stomatal in each sample. The results were obtained from three biological experiments with three technical repetitions.

### 4.8. RNA Sequencing Analysis of CsSDG36 Overexpression Line 2 and Wild Type

In order to study the gene expression dynamics of overexpressed *Arabidopsis thaliana* strains, the leaf and root samples of *Arabidopsis thaliana* overexpression line 2 and wild type were collected for RNA isolation using the rapid RNA isolation kit (Beijing Huayueyang Biotechnology Co., Ltd., Beijing, China) and the quality of the RNA was tested. The qualified RNA samples were embedded in dry ice and sent to Tianjin Novogene Co., LTD. Tianjin For the library construction process, RNA samples were pooled, respectively, according to the effective concentration and the amount of target data for RNA-seq. After statistics, quality control, assembly analysis and functional annotation for the raw transcriptome data, the obtained transcripts were subjected to differential gene screening, GO enrichment and KEGG pathway analysis. The results were obtained from three biological experiments.

### 4.9. qRT-PCR Analysis

Total plant RNA was extracted using the “rapid RNA isolation kit” (Beijing Huayueyang Biotechnology Co., Ltd., Beijing, China), and reverse transcription was performed using the TRUEscript RT Kit (+gDNA Eraser) reagent from Beijing Adlai Biotechnology Co., Ltd., Beijing, China. Agarose gel electrophoresis was used to detect the PCR products. The qRT-PCR reaction was performed using the “2× SYBR Green qPCR Mix (with 100 × ROX)” reagent from Beijing Adlet Biotechnology Co., Ltd., Beijing, China. The PCR cycle was performed with a three-step method, with *GADPH* as the reference gene, using the primers shown in Table 3. The relative expression was determined by the CT (ΔΔCT) method: relative expression of genes = 2^−Δ CT^ = 2^−(CT target gene − CT reference gene)^. The results were obtained from three biological experiments with three technical repetitions.

### 4.10. Statistical Analysis

All the data and error bars were calculated using three independent experiments. The *t*-test was used to determine the significant difference between the two samples. The results were considered statistically significant at *p* < 0.05.

## Figures and Tables

**Table 1:** Bioinformatic analysis of CsSDG36 protein.

Sequence Analysis	
Molecular mass	118,249.89 Da
Theoretical isoelectric point (PI)	8.87
Positively changed residues (Asp + Glu)	121
Negatively charged residues (Arg + Lys)	153
Unstable coefficient	45.41 (unstable)
Aliphatic amino acid	85.5%
Aromatic amino acid	8.0%
Heterocyclic amino acid	6.5%
Acidic amino acid	11.6%
Basic amino acid	16.9%
GRAVY	−0.440 (hydrophilic)
Transmembrane structure	Do not cross the membrane
Subcellular localization	nucleus

**Table 2:** Primer sequence.

Gene Name	Primer Sequence (5′-3′)
*CsSDG36*-F	AAGAGGTGGTTGTGATTGGAGAGG
*CsSDG36*-R	AGTAGAGGGTGGTTGGGTTAGTGC
TYXL+*CsSDG36*-F	TATGACCATGATTACGAATTCAAGAGGTGGTTGTGATTGGAGAGG
TYXL+*CsSDG36*-R	ACGACGGCCAGTGCCAAGCTTAGTAGAGGGTGGTTGGGTTAGTGC

**Table 3:** Primers used for qRT-PCR analysis.

Gene Name	Gene ID	Primer Sequence
*BASL*	AT5G60880	CGATGTGGTTAAAGAGGGTA
		CCGCTAGATTTATCAGAGGC
*SBT1.2*	AT1G04110	ACAGGAGGAGATAAAGGAAGT
		ACCGTGGCATTAACATAAGC
*EPF2*	AT1G34245	GCGTGTTCTTTGGTCGTTAA
		CGTGATAGTATCTCCCTCTGC
*TCX3*	AT3G22760	CGGGAAAGATTCAGGACAAA
		GCGAGTAGCCAGGACAACAT
*CHAL*	AT2G30370	CTACTCTTCTTCGTCCTCTGTG
		TACTGTCCTTGTCCTCGTGT
*TMM*	AT1G80080	AAGATCGCTTGATTTGAGTGG
		AAGACGGGAATGGACCTGAT
*SPCH*	AT5G53210	TCATAGGAGGAGTTGTGGAG
		CTGTGGGATGAGTGGTAGTT
*ERL1*	AT5G62230	CTTGCCAACAACCGTTTAGT
		AGAAGTTATTGCCAGACAGA
*EPFL9*	AT4G12970	ATGAAGCATGAAATGATGAACA
		GGGTCATTTCCTTCGACTG
*AtGADPH*	AT3G04120	TTGGTGACAACAGGTCAAGCA
		AAACTTGTCGCTCAATGCAATC

## Data Availability

Transcriptome data are available at NCBI database (PRJNA728245).

## Supplementary Materials

The following are available online at https://www.mdpi.com/article/10.3390/ijms22105064/s1, Figure S1: The hydrophilic and hydrophobic map of the CsSDG36 protein. Figure S2: The dry weight of single seedling of over-expression lines and wild type after 4-week normal cultivation. The results were obtained from three biological experiments. Figure S3: Transcriptome analysis. Gene ontology (GO) analysis of leaves (A) and roots (B). The results were obtained from three biological experiments. Table S1: GO enrichment of DEGs (OE2L vs. WTL). Table S2: GO enrichment of DEGs (OE2R vs. WTR).
