# Supplementary material for: Ectopic Overexpression of Histone H3K4 Methyltransferase CsSDG36 from Tea Plant Decreases Hyperosmotic Stress Tolerance in Arabidopsis thaliana
Source: Int J Mol Sci. 2021 May 11;22(10):5064. doi: 10.3390/ijms22105064 (PMC8150943; doi:10.3390/ijms22105064)
Supplement: Supplementary file 1 [file ijms-22-05064-s001.zip › Supplimentary figures.pdf]

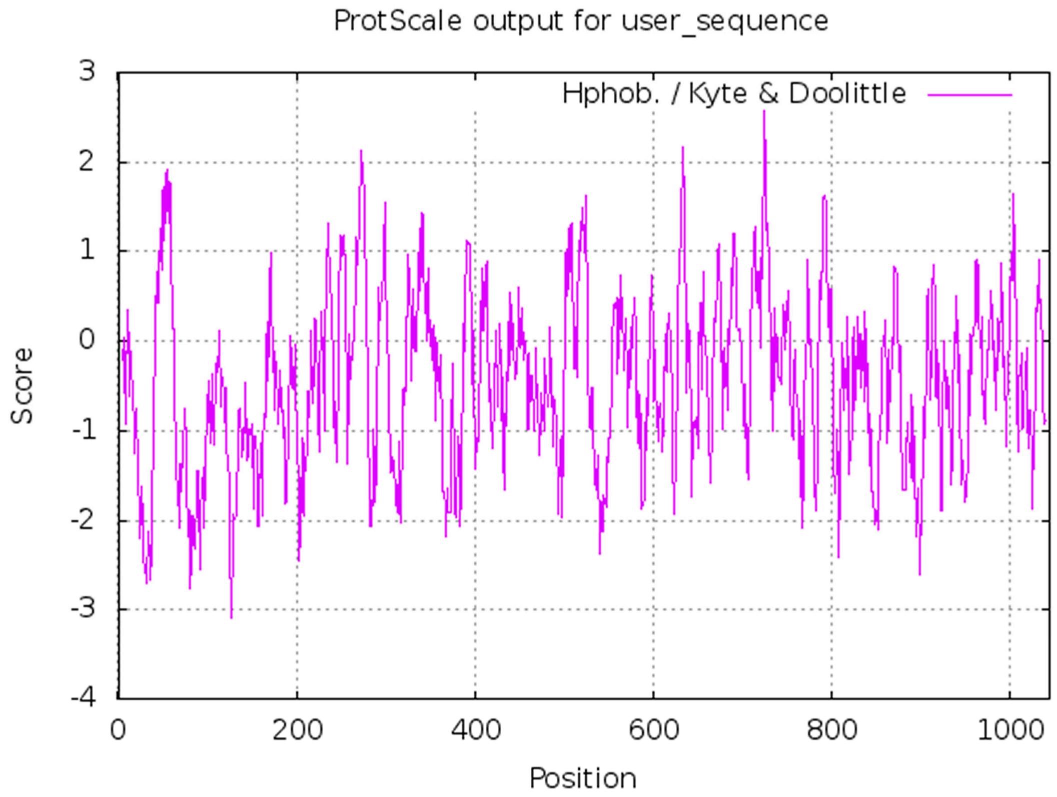

Figure S1: The hydrophilic and hydrophobic map of the CsSDG36 protein.

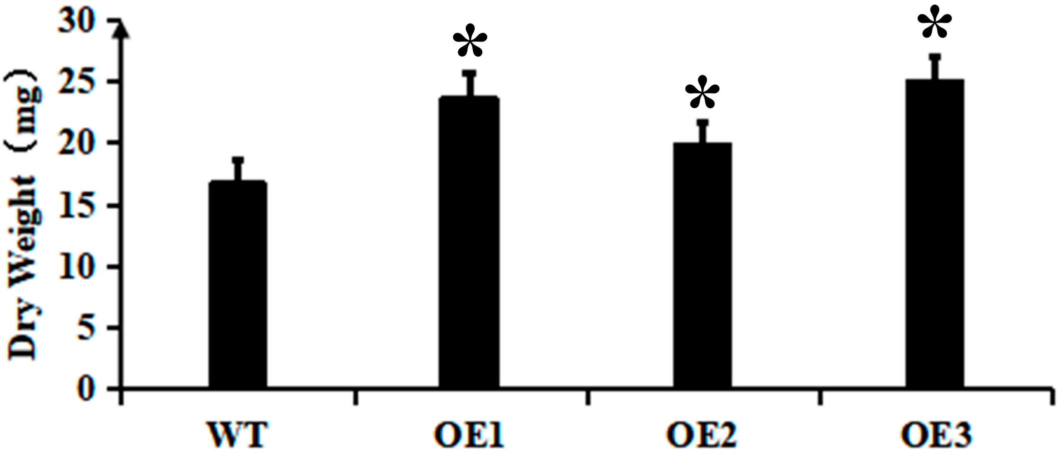

Figure S2. The dry weight of single seedling of over-expression lines and wild type after 4-week normal cultivation. The results were obtained from three biological experiments.

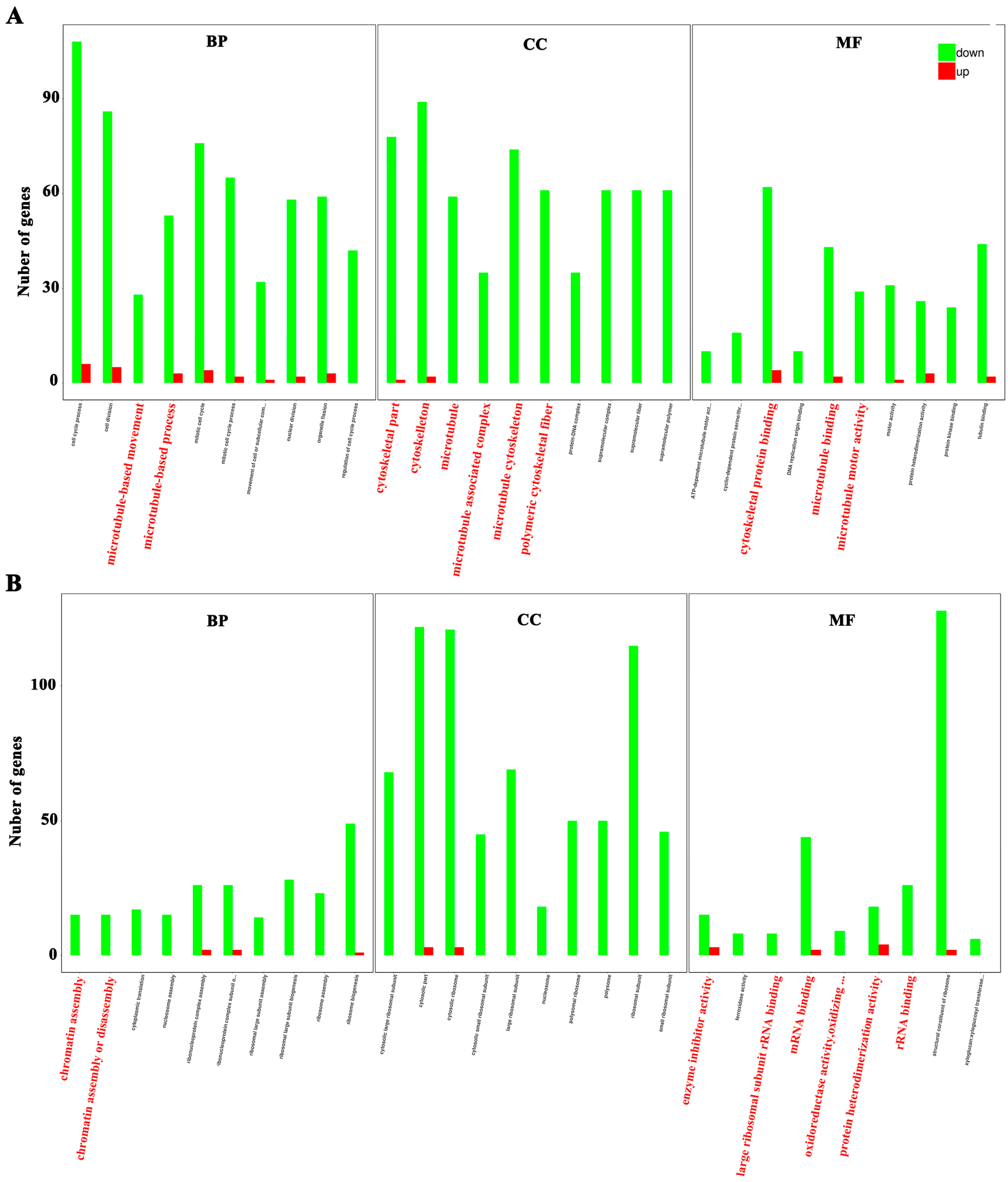

Figure S3. Transcriptome analysis. Gene ontology (GO) analysis of leaves (A) and roots (B). The results were obtained from three biological experiments.
