# Supplementary figures and images for: Ectopic Overexpression of Histone H3K4 Methyltransferase CsSDG36 from Tea Plant Decreases Hyperosmotic Stress Tolerance in Arabidopsis thaliana
Source: Int J Mol Sci. 2021 May 11;22(10):5064. doi: 10.3390/ijms22105064 (PMC8150943; doi:10.3390/ijms22105064)

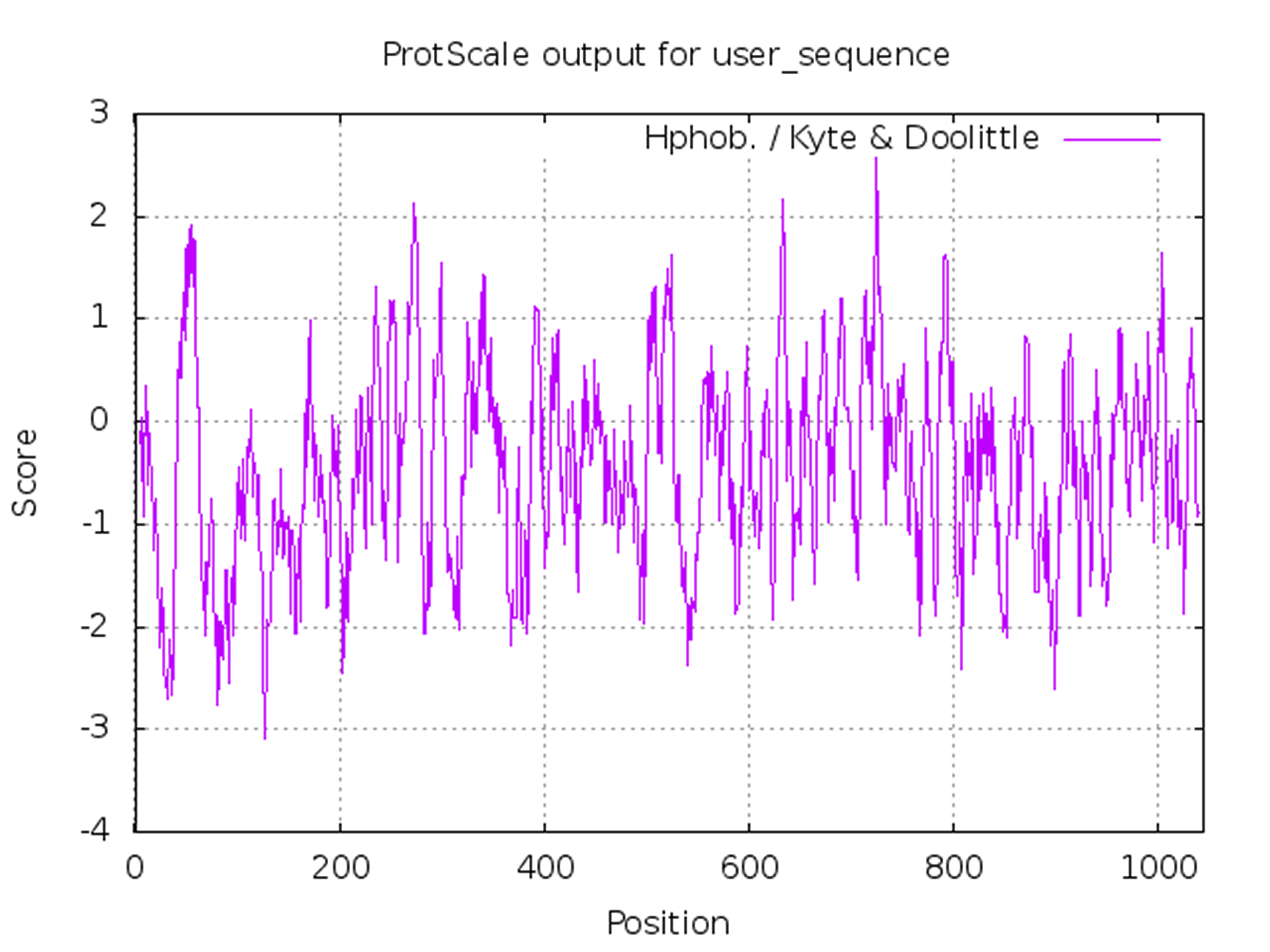

Supplement: Supplementary file 1 [file ijms-22-05064-s001.zip › Figure S1.tif]

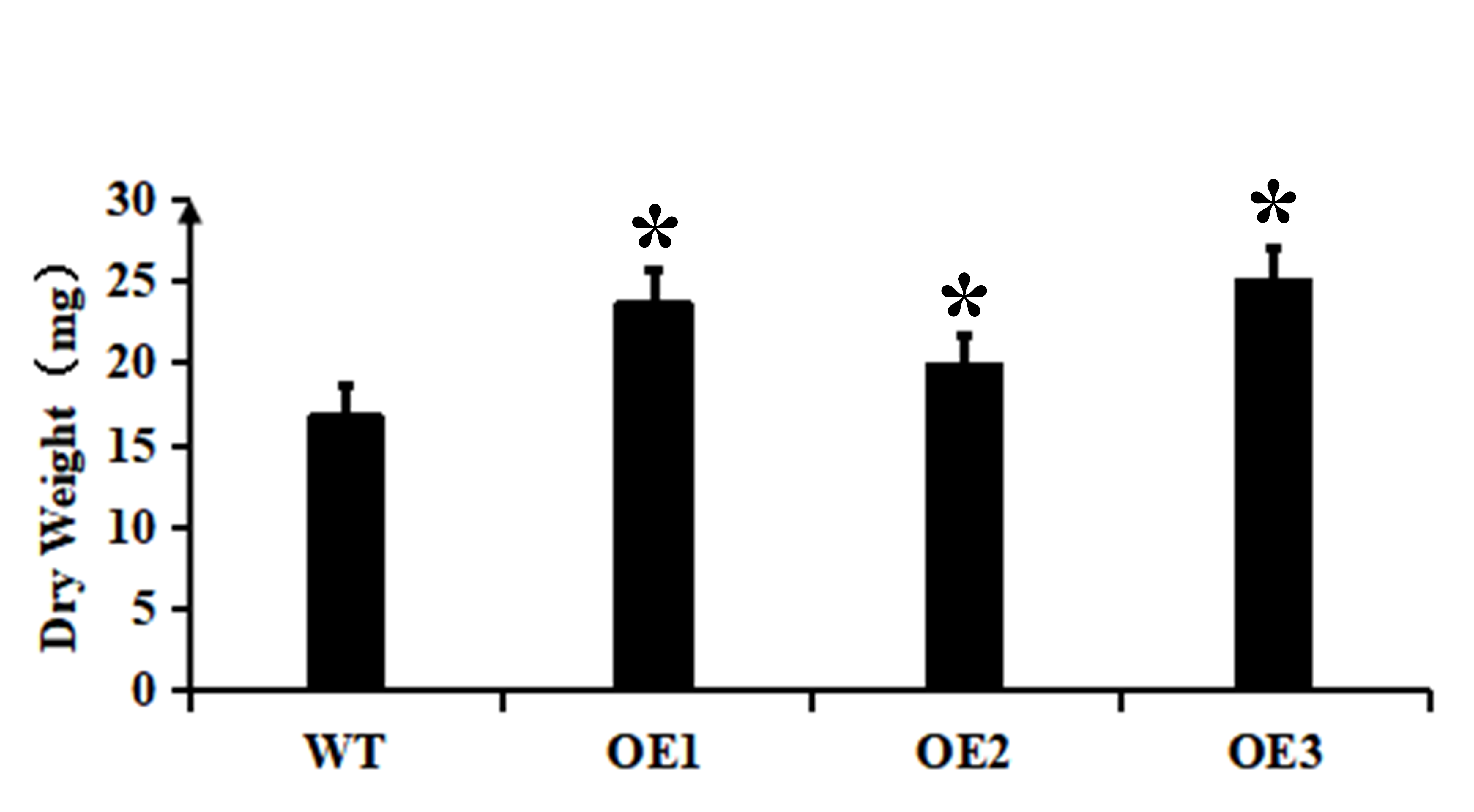

Supplement: Supplementary file 1 [file ijms-22-05064-s001.zip › Figure S2.tif]

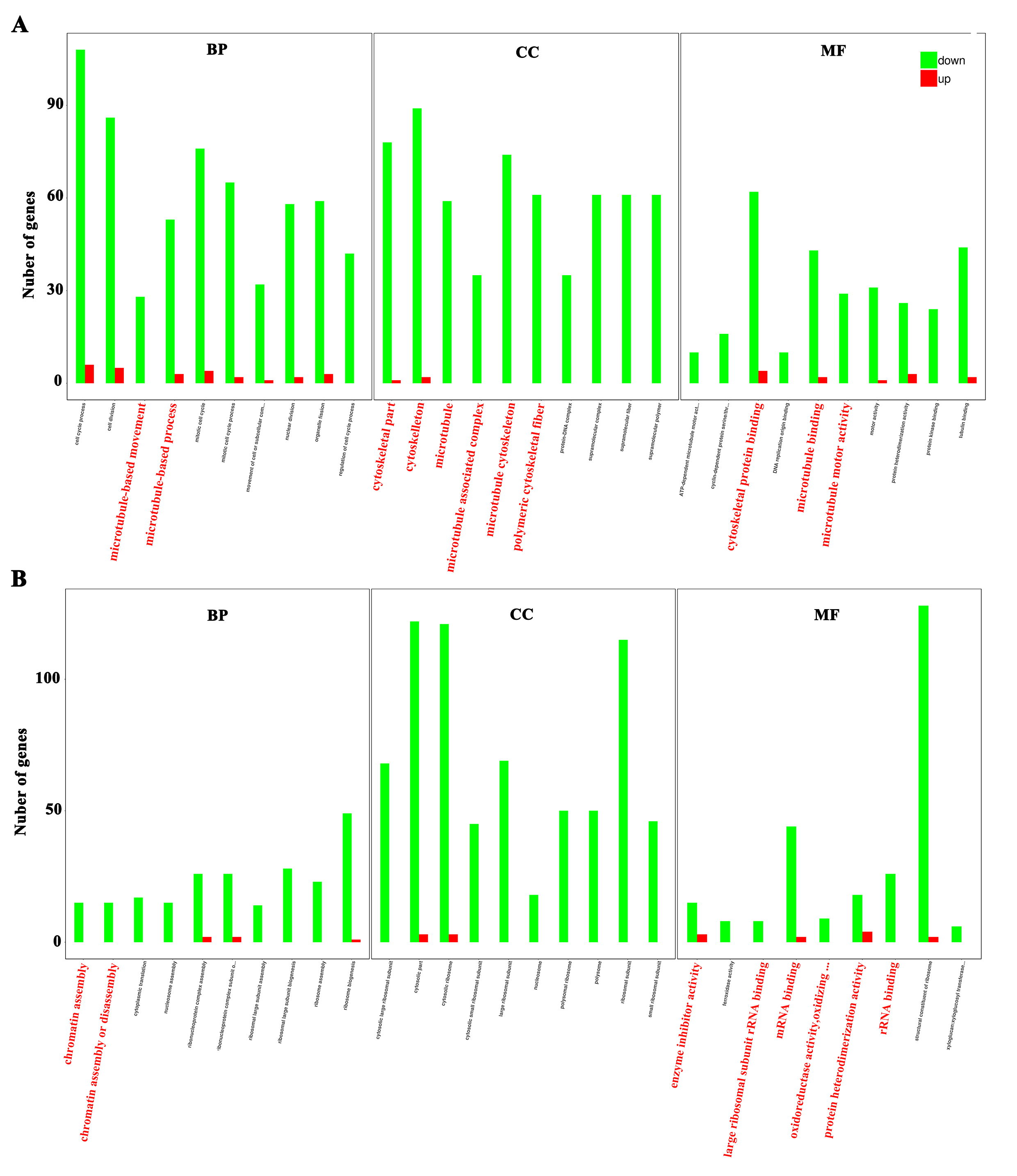

Supplement: Supplementary file 1 [file ijms-22-05064-s001.zip › Figure S3.tif]
